# Prognostic Value of BRAF, Programmed Cell Death 1 (PD1), and PD Ligand 1 (PDL1) Protein Expression in Colon Adenocarcinoma

**DOI:** 10.3390/diagnostics13020237

**Published:** 2023-01-09

**Authors:** Afaf T. Ibrahiem, Entsar Eladl, Eman A. Toraih, Manal S. Fawzy, Khaled Abdelwahab, Khaled Elnaghi, Ziad Emarah, Aly A. M. Shaalan, Ziad Ehab, Nahed A. Soliman

**Affiliations:** 1 Department of Pathology, Faculty of Medicine, Mansoura University, Mansoura 35516, Egypt; 2 Department of Laboratory Medicine and pathobiology, University of Toronto, Toronto, ON M5S 1A8, Canada; 3 Division of Endocrine and Oncologic Surgery, Department of Surgery, School of Medicine, Tulane University, New Orleans, LA 70112, USA; 4 Medical Genetics Unit, Histology and Cell Biology Department, Faculty of Medicine, Suez Canal University, Ismailia 41522, Egypt; 5 Department of Medical Biochemistry and Molecular Biology, Faculty of Medicine, Suez Canal University, Ismailia 41522, Egypt; 6 Department of Biochemistry, Faculty of Medicine, Northern Border University, Arar 73213, Saudi Arabia; 7 Surgical Oncology Department, Faculty of Medicine, Mansoura University, Mansoura 35516, Egypt; 8 Medical Oncology Unit, Oncology Center, Internal Medicine Department, Faculty of Medicine, Mansoura University, Mansoura 35516, Egypt; 9 Oncology Center, King Abdullah Medical City, Makkah 24246, Saudi Arabia; 10 Department of Anatomy, Faculty of Medicine, Jazan University, Jazan 82621, Saudi Arabia; 11 Department of Histology and Cell Biology, Faculty of Medicine, Suez Canal University, Ismailia 41522, Egypt; 12 Faculty of Medicine, Mansoura University, Mansoura 21955, Egypt; 13 Department of Pathology, Faculty of Medicine, Helwan University, Cairo 11795, Egypt

**Keywords:** BRAF, colorectal cancer, protein expression, PD1, PDL1, prognosis, survival

## Abstract

Patients with colorectal cancer in different stages show variable outcomes/therapeutic responses due to their distinct tumoral biomarkers and biological features. In this sense, this study aimed to explore the prognostic utility of BRAF, programmed death-1 (PD1), and its ligand (PDL1) protein signatures in colon adenocarcinoma. The selected protein markers were explored in 64 archived primary colon adenocarcinomas in relation to clinicopathological features. BRAF overexpression was found in 39% of the cases and was significantly associated with grade 3, N1, advanced Dukes stage, presence of relapse, and shorter overall survival (OS). PD1 expression in the infiltrating immune cells (IICs) exhibited significant association with T2/T3, N0/M0, early Dukes stage, and absence of relapse. PDL1 expression in IICs is significantly associated with advanced nodal stage/distant metastasis, advanced Dukes stage, and shorter OS. Meanwhile, PDL1 expression in neoplastic cells (NC) was associated with the advanced lymph node/Dukes stage. A positive combined expression pattern of PDL1 in NC/IICs was associated with poor prognostic indices. Tumor PDL1 expression can be an independent predictor of OS and DFS. The multivariate analyses revealed that short OS was independently associated with the RT side location of the tumor, PD1 expression in stromal IICs, and PDL1 expression in NC. In conclusion, overexpression of BRAF in colon adenocarcinoma is considered a poor prognostic pathological marker. In addition, PDL1 expression in NC is considered an independent prognostic factor for DFS/OS. Combined immunohistochemical assessment for BRAF and PD1/PDL1 protein expressions in colon adenocarcinoma might be beneficial for selecting patients for future targeted therapy.

## 1. Introduction

According to GLOBOCAN 2018 data, colorectal cancer (CRC) is the fourth most common cancer diagnosed globally [1,2]. Although cancer screening programs and improved pre- and post-operative care have reduced mortality associated with CRC, it is still the third cause of cancer-related deaths worldwide [3]. The 5-year survival rate of patients with metastatic disease is still less than 10%, which is probably due to limitations in early diagnoses and the lack of specific markers to determine tumor development or the patient’s prognosis [4].

Cancer management, including CRC, has witnessed some of the most significant advances in adjuvant treatment, target therapy, immunotherapy, and follow-up strategies [5,6,7,8,9,10]. Recently, immunotherapy has been considered an effective treatment for many types of cancers, such as as gastric carcinoma, malignant melanomas, non-small cell lung cancer, renal cell carcinomas, and bladder carcinomas [11,12,13,14,15]. The most important therapy is that which targets the programmed death 1 (PD1)/PD-ligand 1 (L1) pathway [16]. This pathway is stimulated by the interaction between PDL-1 on tumor cells and the PD1 expressed on activated T (CD8+) cells, B cells, and natural killer cells, resulting in T cells apoptosis with subsequent downregulation of the antitumor responses of T cells [17,18,19,20]. In addition, PDL1 expressed by tumor cells and their related stromal cells can be stabilized by tumor necrosis factor alpha (TNF-α) and causes the suppression of antitumor immunity [21].

Aberrant PD1 and PDL1 expression were reported in several types of cancers, including skin cancer, gastric cancer, pancreatic cancer, and breast cancer [19,22,23]. In CRC, PDL1 expression is implicated in tumorigenesis, and its prognostic importance is not fully clarified [4].

Alteration in the BRAF pathway is reported in up to 20% of colorectal tumorigenesis, as it results in uncontrolled cellular growth [24]. It is implicated in the serrated neoplastic pathway, aggressive phenotype, and poor prognosis in stage IV CRC [3,4]. BRAF inhibitors appear to reverse some tumor-associated immune-suppressive signals, and the immune-stimulatory effects observed in response to treatments subside with disease progression [25]. However, the targeted BRAF therapy alone is not very effective, and it was reported to be associated with the persistence of a high level of tumor PDL1 expression [26,27].

For these considerations, this study aimed to assess the prognostic value of PD1/PD-L1 and BRAF proteins expression in colorectal carcinoma by immunohistochemical analysis. This may provide hope for combined BRAF inhibitors and immunotherapy application in such cases in the near future.

## 2. Materials and Methods

### 2.1. Patients

One hundred consecutive cases of primary colon adenocarcinomas were collected retrospectively from Mansoura University, Faculty of Medicine, Oncology Center, Egypt, between July 2014 and June 2017. All patients underwent curative R0 resections. Patients with double malignancies, patients who received previous chemotherapy or radiotherapy, and patients with no available follow-up data were excluded. All patients were followed up regularly at three, six, and 12-month intervals following the guidelines of the German tumor centers (completeness index of 0.96) [28].

**Figure 1:**
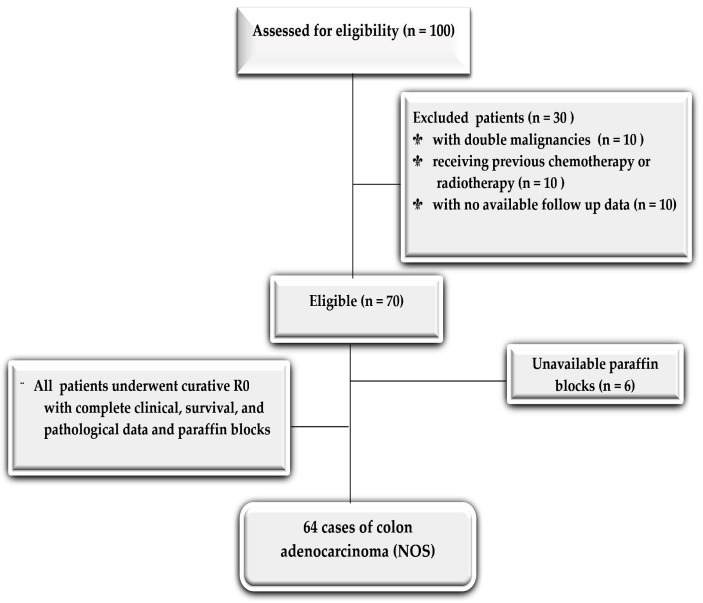
Consort flow diagram for the selection of the study cohort.

Only 64 eligible patients had complete clinical, survival, and pathological data with paraffin blocks (Figure 1). The study was approved by the regional Ethics committee for the Faculty of Medicine, Mansoura University, Egypt (approval no. R.21.02.1198.R1). The demographic data, such as the patient’s age, sex, tumor location, and post-operative course (recurrence and survival), were obtained from the patient’s medical records.

### 2.2. Histopathology

Paraffin-embedded blocks of tumor tissue, as well as adjacent normal colon tissues from the patients, were retrieved from archives of pathology. Serial sections from each specimen were stained with H&E for histological evaluation. Two pathologists reviewed the histopathological features of each slide according to WHO classification [29]. TNM staging and Dukes staging of each tumor were reviewed according to Akkoca et al. [30].

### 2.3. Immunohistochemical (IHC) Analysis and Interpretation

Tissue sections were dewaxed, rehydrated, and washed in phosphate-buffered saline 1× (PBS; Lonza, Verviers, Belgium). Epitope retrieval was performed by treating the slides in a PT Link (Dako, Agilent Technologies, Santa Clara, CA, United States) containing acid or basic solution (as appropriate), preheated to 97 °C for 30 min. Next, endogenous peroxidase was inhibited with a peroxidase-blocking solution (Dako, Agilent Technologies, Santa Clara, CA, United States) for 5 min. Afterward, sections were immunostained with the following primary antibodies: anti-PDL1 (Clone, YPA1638, 1:50, Biospes, Chongqing Biospes Co., Ltd., Chongqing, China), anti-PD1 (Clone, YPA1637, 1:50, Biospes, Chongqing Biospes Co., Ltd., Chongqing, China), and anti-BRAF V (Catalog No. IHC-00607, GeneID 673, Isotype IgG, dilution 1:50, BETHYL laboratory, Montgomery, TX, USA). Tonsil tissue and breast cancer tissue were applied as positive controls for PD1/PDL1 and BRAF antibodies, respectively (following the manufacturer’s guide). The sections were counterstained for 3 min with Meyer’s hematoxylin, then mounted. Negative controls were obtained by omitting the primary antibodies.

The stain was interpreted independently by two observers blinded to the clinical outcome. The Hercept Test scoring system was used to detect the staining score of both PDL1 and PD1 in the infiltrating immune cells and PDL1 in tumor cells. For PDL1 expression of the tumor cells, the intensity of the stain was scored as 0 (no staining), 1 (light yellow), 2 (brown), and 3 (deep brown). The number of stained cells per 100 was scored as 1 (≤10%), 2 (10%~50%), and 3 (≥50%). High PDL1 expression was detected when the product of the staining strength score multiplied and the number of stained cells per 100 cells was no less than three. With regard to immune cell-specific PDL1/PD1 expressions, the percentage of stained cells per 100 cells were detected and categorized as 0–9%, 10–49%, and 50–100% stained immune cells [31].

For BRAF staining, the intensity of the anti-BRAF antibody in tumor cells was recorded on a 0–3 scale. The expression was mainly cytoplasmic with nuclear staining in cases with strong and moderate cytoplasmic staining. Strong cytoplasmic with or without nuclear staining was scored as 3, moderate cytoplasmic staining with or without nuclear staining as 2, weak cytoplasmic staining as 1, and the absence of staining was scored as 0. In addition, any nuclear staining and the percentage of tumor cells stained positive with anti-BRAF antibodies were recorded. The cases were scored as dysregulated BRAF protein expression if > 80% of tumor cells expressed diffuse uniform unequivocal strong or moderate cytoplasmic staining with or without nuclear staining. However, they were scored negative for dysregulated BRAF expression if they showed no staining or weak, cytoplasmic, non-granular, uniform staining (stain intensity <80%). The cases with staining of isolated tumor cells in a tumor and those who showed no staining were also considered negative cases for the dysregulated BRAF expression. The cases were scored as equivocal if they displayed ambiguous, heterogeneous, non-uniform cytoplasmic staining in tumor cells with or without nuclear staining [24].

### 2.4. Combined Expression Patterns of PDL1 in Neoplastic Cells (NC) and Infiltrating Immune Cells (IIC)

The study cases were categorized into four groups according to the combined expression patterns of PDL1 in neoplastic cells (NC) and infiltrating immune cells (IIC), a method which is validated by Valentini et al. 2018 (22). Group A (NC−/IIC−) which was negative in NCs and IICs; Group B (NC+/IIC−) which was positive only in neoplastic cells; Group C (NC−/IIC+) was positive only in IICs; Group D (NC+/IIC+) was positive both in NCs and IICs. The expression pattern of each marker in both NC and IIC was tested for association with clinicopathological parameters.

### 2.5. Statistical Analysis

Data were analyzed using R version 3.5.1 and SPSS version 23.0. Chi-square and Fisher’s Exact tests were applied for qualitative variables (when appropriate), while student’s-t and Mann–Whitney U tests were employed for continuous attributes. *p*-value ≤ 0.05 was set to be significant.

Patients were grouped into four groups based on PDL1 expression and location. The association of the expression patterns and the clinicopathological parameters were tested using the Kruskal–Wallis test. Differences in overall survival (OS) and disease-free survival (DFS) between groups were assessed using the log-rank test, and Kaplan–Meier curves were plotted. Univariate and multivariate Cox proportional hazard regression analyses were performed to calculate the hazard ratio (HR) and 95% confidence interval (CI).

## 3. Results

### 3.1. The Clinicopathological Characteristics of the Studied Cases

Table 1 summarizes the clinicopathological features of the studied 64 cases of colorectal adenocarcinoma. The mean age at diagnosis was 57.3 ± 12.5 years. Twenty-nine cases (45.3%) were ≤ 55 years, and twenty-nine cases (45.3%) were females. In 52 cases, the tumor was located in the ascending colon (81.3%), and, in the other 12 cases, were located in the descending colon and rectum (18.8%). About 38 cases (59.4%) were grade II, and 17 cases (26.5%) were grade III. The most prevalent T stages were T3 (40 cases; 62.5%) and T2 (15 cases; 23.4%). The nodal stages varied from N0: 37 (57.8%), N1: 24 (37.5%), and N2: 3 (4.7%). Most cases had no metastasis; only five (7.8%) had distant metastasis. The studied cases were in Dukes stage A: 10 (15.6%), B: 26 (40.6%), C: 23 (35.9%), and D: 5 (7.8%). During the follow-up period (34 ± 20 months), nearly 61% of the cases had not relapsed, while 45.3% died.

### 3.2. BRAF Protein Expression and Association with Clinicopathological Prognostic Factors of Colon Adenocarcinoma

**Figure 2:**
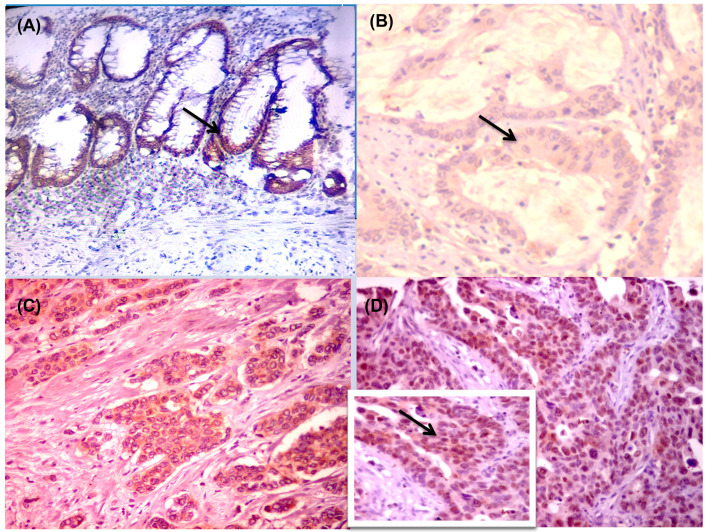
Immunohistochemical staining of colonic adenocarcinoma for BRAF protein showed aberrant cytoplasmic expression in the adjacent non-neoplastic colonic mucosa ((**A**) × 200). Colonic adenocarcinoma did not express staining for BRAF protein ((**B**) × 400), colonic adenocarcinoma expressed diffuse strong cytoplasmic staining ((**C**) × 200), while other colonic adenocarcinoma showed diffuse strong cytoplasmic staining associated nuclear staining for BRAF protein ((**D**) × 400).

BRAF protein expression was considered positive in 25 cases (39.1%). The staining was cytoplasmic with or without nuclear staining. Adjacent non-neoplastic colorectal tissue showed scattered nuclear staining in the mucosa and/or cytoplasmic staining in smooth muscle (Figure 2).

As regards the association of BRAF protein expression with the clinicopathological characteristics of the studied colonic adenocarcinoma cases, using a two-sided Chi-square test, there were significant associations with grade III (*p* = 0.04), N1 (*p* = 0.00), advanced Dukes stage (C-D) (*p* = 0.02), presence of relapse (*p* = 0.02), and shorter overall survival (OS; *p* = 0.00). At the same time, BRAF protein expression was not associated with the presence of lymphovascular invasion (LVI; *p* = 0.06) (Table 2).

### 3.3. Expression of PD1 and PDL1 and Association with Clinicopathological Prognostic Factors of Colon Adenocarcinomas

**Figure 3:**
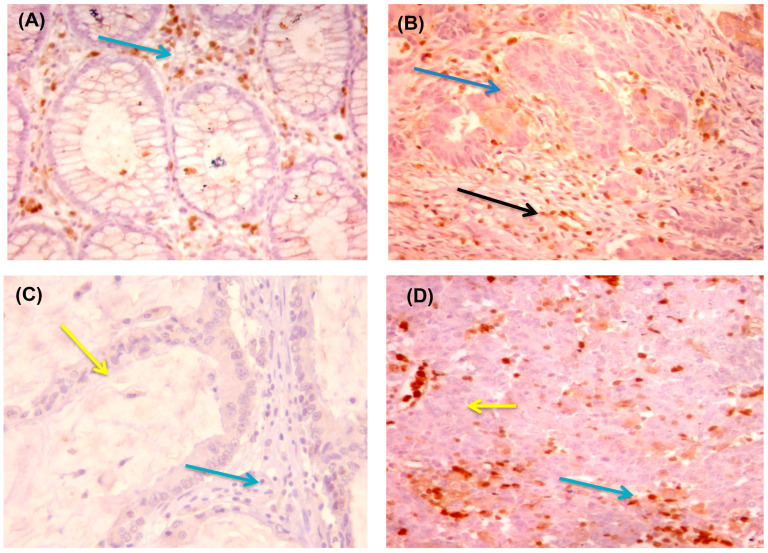
Immunohistochemical staining of colonic adenocarcinoma for PD1. Adjacent non-neoplastic colonic mucosa showed positive staining of the mucosal lymphocytes for PD1 ((**A**) ×400), G1 adenocarcinoma showed positive staining of intratumoral immune cells for PD1 (blue arrow) with no expression in tumor cells (yellow arrow) ((**B**) × 200 and (**C**) × 400). In addition, G3 adenocarcinoma showed expression of PD1 on intratumoral immune cells (blue arrow) with negative expressions on tumor cells (yellow arrow) ((**D**) × 400). N.B., as the lymphocytes have little cytoplasm, the staining appears mainly cytoplasmic.

In adjacent non-neoplastic colonic mucosa epithelial cells, PD1 and PDL1 proteins showed no staining. The pattern of PD1 and PDL1 expression in neoplastic cells was either focal or diffuse, with a predominance of the focal pattern, particularly along with the tumor–stromal interface. While in infiltrating immune cells (IIC), a diffuse positive pattern was prevalent. Among the 64 patients, 41 (64%) had PD1 overexpression in IIC (Table 3, Figure 3).

**Figure 4:**
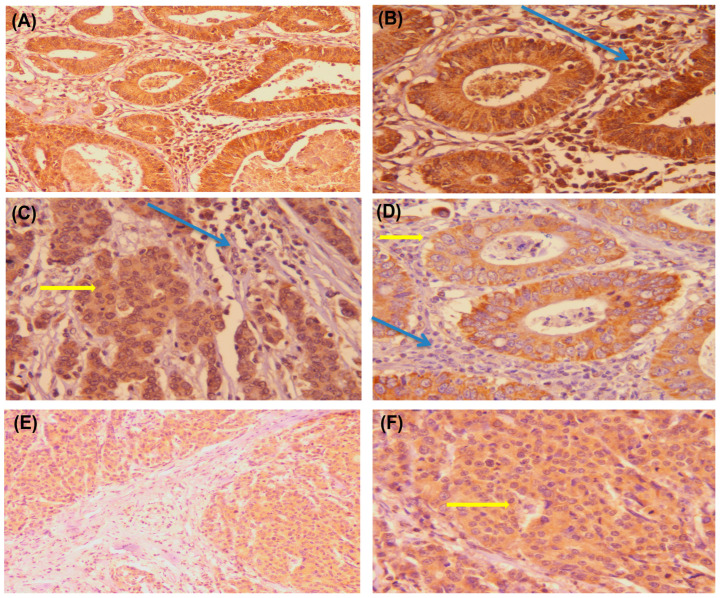
Immunohistochemical staining of colonic adenocarcinoma for PDL1. Positive expressions of PDL1 on tumor cells (yellow arrow) and intertumoral lymphocytes in G1 colonic adenocarcinoma (blue arrow) were observed ((**A**) ×200 and (**B**) × 400). G2 adenocarcinoma showed moderate expression of PDL1 on intratumor immune cells (blue arrow) and tumor cells (yellow arrow) (**C**) × 400). The expression is mainly cytoplasmic; however, in the photo ((**D**) × 400), the expression of PDL1 in tumor cells was mainly membranous (yellow arrow) with no expression on intratumor immune cells (blue arrow). In addition, G3 adenocarcinoma showed moderate expression of PDL1 on tumor cells (yellow arrow in panel (**F**)) ((**E**) × 200 and (**F**) × 400).

PDL1 overexpression in NC was detected in 34 cases (53.1%), and PDL1 overexpression in IICs was detected in 33 cases (51.6%) (Table 1). Staining was predominantly localized in the cellular membrane with diffuse faint intracellular expression (Table 1, Figure 4).

Regarding PDL1 overexpression in NC and IIC association with the clinicopathological characteristics, using a two-sided Chi-square test, there were significant associations with the advanced lymph node stage (*p* = 0.00, 0.000) and advanced Dukes stage (*p* = 0.000, 0.000). In addition, PDL1 overexpression in IICs exhibited a significant association with the presence of distant metastasis (*p* = 0.01) and shorter OS (*p* = 0.01). While PD1 overexpression in IICs exhibited significant association with T2/T3 (*p* = 0.002), N0 (*p* = 0.00), M0 (*p* = 0.00), early Dukes stage (*p* = 0.00), and absence of relapse (*p* = 0.00) (Table 3).

### 3.4. Combined Expression Patterns of PDL1 in Neoplastic and Infiltrating Immune Cells, and Association with the Clinicopathological Prognostic Factors of Colon Adenocarcinomas

The combined expression pattern of PDL1 includes four groups. Group A (NC−/IIC−), Group B (NC+/IIC−), Group C (NC−/IIC+), and Group D (NC+/IIC+). The studied cases include 18 cases (group A), 17 cases (group B), 10 cases (group C), and 19 cases (group D). Combined expression pattern group D was significantly associated with poor prognostic parameters, including advanced nodal stage (*p* = 0.002), advanced Dukes tumor stage (*p* = 0.01), and shorter OS (*p* = 0.05) using the Kruskal–Wallis test (Table 4).

### 3.5. Correlation between BRAF, PD1, and the PDL1 Protein Expressions

By testing the way of linking the expression pattern of the three markers using the Spearman Bivariate correlation test, BRAF protein expression positivity did not correlate with either IICs PD1, NC PDL1, IIC PDL1, or the combined pattern of PDL1. Only NC PDL1 and IIC PDL1 show a considerable positive correlation to the combined pattern of PDL1 expression (r: 0.591 and 0.895, respectively (*p* = 0.000 for both)) (Table 5).

### 3.6. Univariate and Multivariate Analyses of Patients’ Clinical Outcomes (Relapse, Overall and Disease-Free Survival) and BRAF, PD1, and PDL1 Expressions

**Figure 5:**
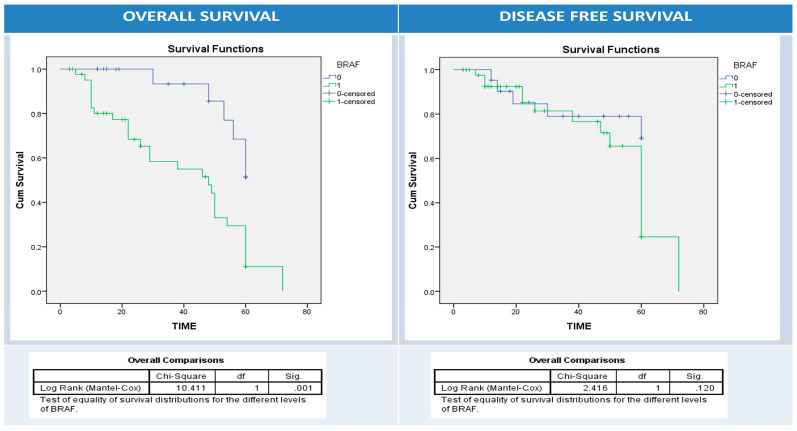
Kaplan–Meier survival curves for overall (right panel) and disease-free survival (left panel) according to BRAF expression (0: blue (-ve BRAF), 1: green (BRAF overexpression)). Log Rank test (Mantel–Cox) was used.

**Figure 6:**
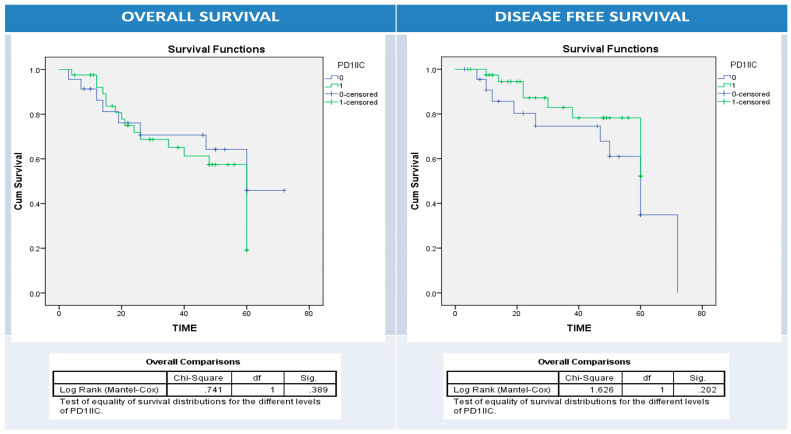
Kaplan–Meier survival curves for overall (right panel) and disease-free survival (left panel) according to IIC PD1 expression (0: blue (-ve PD1 IIC); 1: green (+ve PD1 IIC)). Log Rank test (Mantel–Cox) was used.

**Figure 7:**
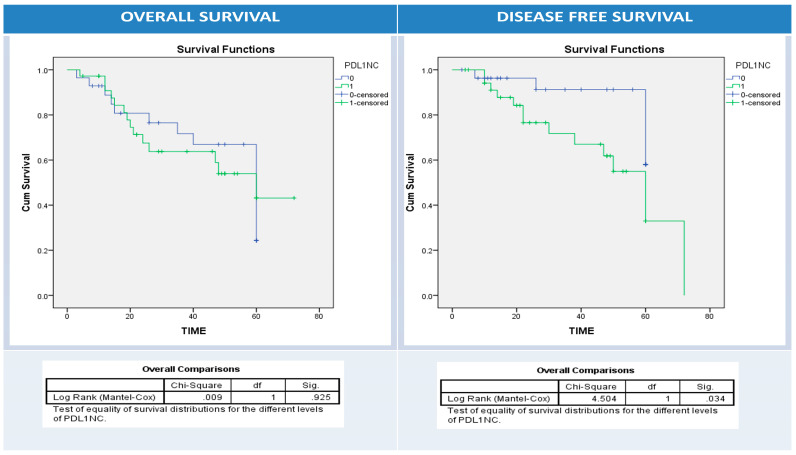
Kaplan–Meier survival curves for overall (right panel) and disease-free survival (left panel) according to neoplastic cells (NC) PDL1 expression (0: blue (-ve PDL1 NC); 1: green (+ve PDL1 NC)). Log Rank test (Mantel–Cox) was used.

**Figure 8:**
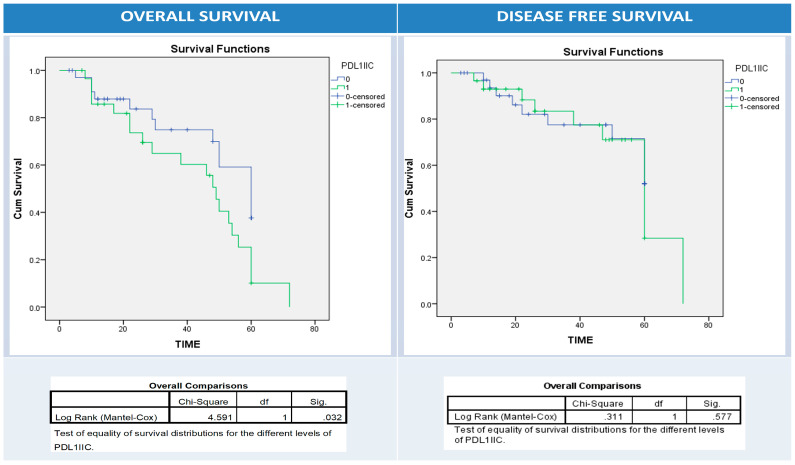
Kaplan–Meier survival curves for overall (right panel) and disease-free survival (left panel) according to intratumor immune cells (IIC) PDL1 expression (0: blue (-ve PDL1 IIC); 1: green (+ve PDL1 IIC)). Log Rank test (Mantel–Cox) was used.

**Figure 9:**
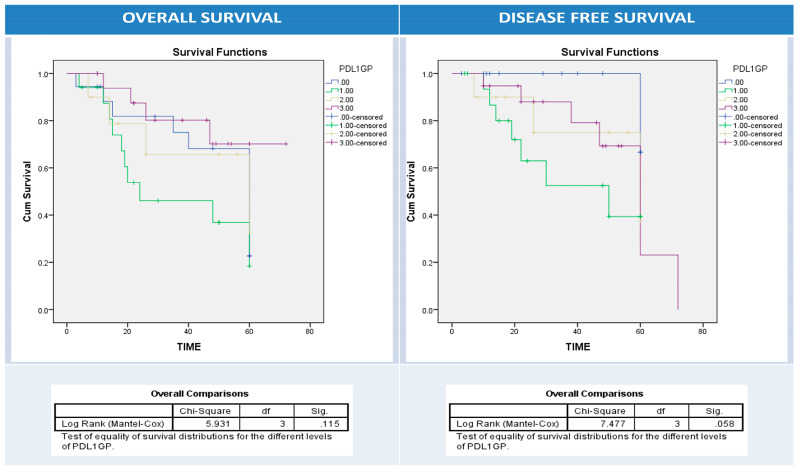
Kaplan–Meier survival curves for overall (right panel) and disease-free survival (left panel) according to combined PDL1 expression pattern (NC−IIC). 0: blue (NC−IIC−); 1.00: green (NC+ IIC); 2.00: yellow (NC−IIC+); 3: purple (NC+IIC+). Log Rank test (Mantel–Cox) was used.

Using univariate survival analysis by Kaplan–Meier curve and the log-rank test, there was no significant difference in disease-free and overall survival rates among patient groups with different BRAF protein, IIC PD1, and PDL1 protein (NC, IIC, or combined pattern) expression patterns apart from BRAF protein expression and overall survival (*p* = 0.001), PDL1 overexpression in NC and disease-free survival (*p* = 0.034), and PDL1 overexpression in IIC and overall survival (*p* = 0.03) (Figure 5, Figure 6, Figure 7, Figure 8 and Figure 9).

Multivariate regression analysis using all clinical and pathological variables failed to detect PDL1 (IIC) and BRAF protein expressions as independent predictors for survival and relapse. However, tumor PDL1 expression can be used as an independent predictor of OS and DFS [OS: HR, 3.250; 95% CI, 1.088–9.713; *p* = 0.035 and DFS: HR, 2.241; 95% CI, 1.003–5.006; *p* = 0.049]. PD1 overexpression in stromal IICs was independently associated with short OS (HR: 0.233, 95% CI: 0.060–0.903, *p* = 0.035). In addition, the right-side location of CRC revealed an independent association with short overall survival (HR: 433.167, 95% CI: 1.437-130573.1, *p* = 0.037) (Table 6).

## 4. Discussion

The recently developed immunotherapeutic strategies have yielded remarkable clinical results in controlling tumor growth in many tumors. It showed the highest response in melanoma, renal cell carcinoma, non-small cell lung carcinoma, and microsatellite instability-high CRC [17,32]. The year 2017 witnessed the first US Food and Drug Administration (FDA) approval of immune checkpoint inhibitor (ICI) immunotherapy for the management of CRC [33]. However, PD1/PDL1 blockade therapy is significantly helpful only in a group of patients, and the others either show resistance or only respond transiently to this therapy [34]. So, identifying resistance mechanisms is crucial to enhance the reach of more responders to this therapy.

Also, accumulating evidence suggested that dysregulated BRAF expression has an immunosuppressive effect and a role in poor response to PD1/PDL1 checkpoint inhibitors targeted immunotherapy. In melanoma, there are mounting data that oncogenic BRAF contributes to immune escape, and several clinical trials combined BRAF inhibitors with immune checkpoint blockade [35]. For these considerations, IHC expression of PD1, PDL1, and BRAF proteins evaluation before therapy may help to determine patients that will benefit from immunotherapy and could be used as a base to design novel combination therapy (immunotherapy and BRAF inhibitors) for CRC.

BRAF is a protein kinase and part of the mitogen-activated protein (MAP) kinase signaling cascade, which involves the transduction of a growth signal from the cell membrane to the nucleus via a chain of protein kinases, and is responsible for cellular proliferation and survival [24]. Detection of BRAF in colon carcinoma has the potential as a prognostic marker and also as treatment target for new BRAF inhibitors, such as vemurafenib [35,36].

Genetic testing is expensive, with high-level laboratory requirements, and needs strict quality control and professional knowledge of molecular detection technology of PCR; hence, it is not conducive to clinical application in areas of limited resources. In contrast, IHC is economical, simple, and feasible. Some studies assessed the feasibility of IHC instead of PCR to detect the mutated BRAF and reported near-to-complete concordance between both techniques in various cancers, including colon carcinomas [37,38,39]. These results support using IHC as a simplified strategy to screen colorectal cancers in clinical practice [40].

Based on previous experience, we used immunohistochemistry to detect dysregulated protein BRAF expression. We followed the previous recommendation to consider diffuse cytoplasmic staining with moderate to intense staining in >80% of tumor cells as positive dysregulated expression; however, weak staining is negative, and heterogenous staining is equivocal [24]; however, using specific anti-BRAF monoclonal antibodies is recommended in future studies to uncover the mutational status of the tested samples.

BRAF protein expression was recorded in 25 cases (39.1%) in the current study with significant association with the advanced grade, lymph node state, Dukes staging, and the occurrence of relapse and short OS. From these results, we concluded that BRAF protein expression in CRC is a poor prognostic marker. These findings agree with the previous studies’ results in which the authors explored the BRAF gene and protein expressions, and found that these expressions were associated with poor prognostic parameters, including the advanced grade and T stage of the tumor, and short OS in the case of protein expression [24,41]. Our findings support the previous studies, which report the significant implication of BRAF in cancer and associate right-sided colon cancer with worse clinical outcomes. BRAF protein is a valuable biomarker for identifying patients who may benefit from a more individualized course of therapy [24,42]. We noticed occasional cytoplasmic and nuclear immunostaining for BRAF in normal mucosa cells (Figure 2). These findings should be interpreted with caution, as incomplete specificity of the anti-BRAF polyclonal antibodies used in the current work and the potential cross-reactivity with other epitopes could play part in this finding, and warrant further confirmatory studies that apply more specific monoclonal antibodies to differentiate mutant vs. wild BRAF protein.

Regarding PD1, positivity in IICs was observed in 64% of cases. PDL1 was expressed in tumor cells in 53.1% of cases, and IICs in 51.6% of cases. Significant correlation between PD1 positivity in IICs and early T stage, negative LN stage, early-stage Dukes, M0, and absence of relapse. These data reflected that immune cell PD1 expression is significantly associated with good prognostic pathological parameters, which agreed with Berntsson et al., who concluded that immune-specific PD1 is significantly associated with lower T and M stages, whatever the location of the tumor, as their study related the side to the prognostic impact of PD1 and PDL1 expressions [43].

As regards PDL1 protein expression in NC and IIC, positive PDL1 expressions in NC showed a significant association with aggressive clinicopathological parameters (advanced nodal stage and Dukes). These data agree with previous studies [4,21]. Juneja et al. confirmed that NC PDL1 could inhibit the antitumor immunity by inactivation of CD8+ TC sensitive to PD1 signaling, and lead to an increase in the aggressiveness of the tumor [21]. Shen et al. concluded a significant association of NC PDL1 expression with advanced cancer stage and lymphatic invasion based upon a meta-analysis of 3481 patients included in 10 studies [4]. However, Berntsson et al. confirmed that neoplastic cell expression of PDL1 was significantly associated with younger age and highly differentiated tumors, but this was in the right-side colon cancer only and not on the left side or in the total cohort study [43].

The IIC PDL1 expression was significantly associated with advanced nodal stage, metastasis, advanced Dukes, and short OS. These results were inconsistent with the Berntsson et al. findings, which confirmed that immune cell expression of PDL1 was significantly associated with lower T, N, and M stages [43]. Such type of difference in the results could be attributed to a large number of Berntsson et al. samples (557 cases) compared to ours (64 cases), the different monoclonal antibodies used, and the different cutoff points of positivity. In addition, the degree of staining intensity was considered in the current work, but not in the Berntsson et al. study [43].

Regarding the pattern of PDL1 expression and tumor immune microenvironment, our study identified four subsets (NC−/IIC−, NC+/IIC−, NC−/IIC+, and NC+/IIC+). We found that combined expression pattern group D was significantly associated with advanced LN and Dukes stage. This classification helps the oncologist select the patient candidate for immune therapy, as the first pattern (NC−/IIC−) will not benefit from the therapy, and the last pattern (group D) will be the ideal candidate for checkpoint inhibitors.

The Spearman Bivariate correlation test revealed a positive correlation between BRAF protein expression scores and positivity. In addition, the test revealed that NC PDL1 is only significantly correlated to the combined pattern of PDL1 expression. However, IIC PDL1 showed a strong positive correlation to the combined pattern of PDL1. BRAF protein expression score or positivity did not correlate with IIC PD1, NC PDL1, IIC PDL1, or the combined pattern of PDL1. These results were consistent with the Berntsson et al. study, both on the correlation between BRAF expression and both PD1 and PDL1 protein expressions [43]. From these results, we can suggest that BRAF protein assessment could be helpful for patients with CRC arranging for combined therapy (immunotherapy and BRAF inhibitors).

Significant heterogeneity in survival outcome characterizes colonic cancer patients with dysregulated BRAF expression due to the complex, and still not entirely fully elucidated, interactions between the clinical, genetic, and epigenetic landscape of BRAF expression [44]. Our study suggested the importance of testing patients for PD1 and PDL1 along with BRAF protein expression evaluation.

Multivariate regression analysis using all clinical and pathological variables failed to detect PD1 (IIC), PDL1 (IIC), and BRAF protein as independent predictors for survival and relapse. However, tumor PDL1 expression can independently predict OS and DFS. This finding was consistent with a recent Wang et al. meta-analysis, in which the meta-regression showed that “PD-L1 expression played a significant role on poor CRC OS (HR  =  1.95, 95% CI (1.92, 3.98)) and DFS (HR  =  2.14, 95% CI (0.73, 4.52))” and could independently predict a poor CRC prognosis [45].

Also, the multivariate analysis revealed that the right-side location of the CRC could be a potential predictor for the short OS. This result is congruent with the Baran et al. report, which emphasized that CRC is not a single entity, but its pathogenesis and treatment response could depend on the anatomical location (i.e., RT vs. LT side). Patients with left-sided CRC showed more response to “5-fluorouracil (5-FU)-based regimes” as one of the adjuvant chemotherapies and also to “anti-epidermal growth factor receptor therapy” as targeted therapy with a better prognosis. In comparison, patients with right-sided CRC showed poor response to conventional chemotherapies, but demonstrated more promising results with immunotherapies, as it is characterized by an increase in the antigenic load [46].

It is noteworthy to consider the limited sample size in this study and the absence of applying a BRAF mutation-specific assay. In this sense, large-scale studies using highly specific (monoclonal antibodies) assays for IHC analysis for the detection of the BRAF mutation are highly recommended. In addition, the adoption of the recent “WHO classification of the digestive system tumors” is warranted in future studies.

## 5. Conclusions

The current study concluded that overexpression of BRAF protein in colorectal carcinoma is a poor prognostic pathological marker. In addition, PDL1 expression in NC is considered an independent prognostic factor for DFS and OS. Our study can suggest that combined immunohistochemical assessment for BRAF protein, PD1, and PDL1 expression in CRC could be beneficial for selecting patients for future combined immunotherapy and BRAF inhibitors.

## Figures and Tables

**Table 1:** The clinicopathological characteristics of the study cases.

Number		64 (100)
Age (years)	Mean ± SD	57.3 ± 12.5
	≤55	29 (45.3)
	>55	35 (54.7)
Sex	F	29 (45.3)
	M	35 (54.7)
Laterality	RT	52 (81.3)
	LT	12 (18.8)
Grade	G1	9 (14.1)
	G2	38 (59.4)
	G3	17 (26.5)
T stage	T1	5 (7.8)
	T2	15 (23.4)
	T3	40 (62.5)
	T4	4 (6.3)
N stage	N0	37 (57.8)
	N1	24 (37.5)
	N2	3 (4.7)
M stage	M0	59 (92.2)
	M1	5 (7.8)
LVI	No	35 (45.3)
	Yes	29 (54.7)
Dukes	A	10 (15.6)
	B	26 (40.6)
	C	23 (35.9)
	D	5 (7.8)
	A+B	35 (54.7)
	C+D	29 (45.3)
Relapse	No	44 (60.9)
	Yes	20 (39.1)
Alive	Dead	35 (45.3)
	Survived	29 (54.7)
BRAF protein	Negative	39 (60.9)
	Positive	25 (39.1)
	Score 0	39 (60.9)
	Score 1	11 (17.2)
	Score 2	9 (14.1)
	Score 3	5 (7.8)
PD1-IIC	Negative	23 (35.9)
	Positive	41 (64.1)
PDL1-NC	Negative	30 (46.9)
	Positive	34 (53.1)
PDL1-IIC	Negative	31 (48.4)
	Positive	33 (51.6)

Data are represented as frequency (percentage) and/or mean ± standard deviation (SD). T; tumor, N; lymph node; M; metastasis (distant); LVI; lymphovascular invasion, IIC = infiltrating immune cells, NC = neoplastic cells.

**Table 2:** Association of BRAF protein expression status and clinicopathological prognostic factors of colorectal cancer.

Variable		Total 64 (100)	BRAF Protein Expression		*p*-Value
			Negative N = 39	Positive N = 25	
Age (y)	Mean		57.9 ± 13.4	58 ± 11	0.9
Age Group	≤55	29	17	12	0.7
	>55	35	22	13	
Sex	F	29	18	11	0.8
	M	35	21	14	
Laterality	Rt	52	30	22	0.5
	Lt	12	9	3	
Grade	G1	9	7	2	**0.04**
	G2	38	25	13	
	G3	17	7	10	
T stage	T1	5	2	3	0.5
	T2	15	8	7	
	T3	40	28	12	
	T4	4	1	3	
N stage	N0	37	31	6	**0.00**
	N1	24	7	17	
	N2	3	1	2	
M stage	M0	59	36	23	0.9
	M1	5	3	2	
LVI	No	35	25	10	0.06
	Yes	29	14	15	
Dukes	A-B	35	28	7	**0.001**
	C-D	29	11	18	
Relapse	No	44	31	13	**0.02**
	Yes	20	8	12	
OS	Dead	35	14	21	**0.00**
	Survived	29	25	4	

Data are presented as frequency (percentage) and/or mean ± standard deviation (SD). Two-sided Chi-square and Kruskal–Wallis tests were used. Bold values indicate a statistically significant *p*-value below 0.05. T: tumor, N: lymph node, M: metastasis (distant); LVI: lymphovascular invasion, OS: overall survival, Rt: Right, Lt: Left.

**Table 3:** Association of PD1 expression in IIC and PDL1 expression in NC and IIC and clinicopathological prognostic factors of colorectal cancer.

Variable		Total	PD1-IIC		*p*	PDL1-NC		*p*	PDL1-IIC		*p*
			Negative	Positive		Negative	Positive		Negative	Positive	
N		64	23	41		28	36		35	29	
Age	M ± SD		58.7 ± 13.6	57.5 ± 12.1	0.7	58.3 ± 13	57.6 ± 12.4	0.8	59.3 ± 12.1	56.3 ± 13.2	0.4
	≤55	29	10	19	0.8	12	17	0.7	14	15	0.3
	>55	35	13	22		16	19		21	14	
Sex	F	29	10	19	0.8	14	15	0.5	16	13	0.9
	M	35	13	22		14	21		19	16	
Location	Rt	47	19	33	0.8	21	31	0.2	28	24	0.7
	Lt	12	4	8		7	5		7	5	
Grade	G1	9	2	7	0.3	5	4	0.1	6	3	
	G2	38	18	20		18	20		21	17	0.3
	G3	17	3	14		5	12		8	9	
T stage	T1	5	0	5	**0.02**	2	3	0.6	1	4	0.5
	T2	15	5	10		8	7		9	6	
	T3	40	14	26		16	24		23	17	
	T4	4	4	0		2	2		2	2	
	T1+T2	17	4	13	0.2	10	7	0.1	10	7	0.6
	T3+T4	47	19	28		18	29		25	22	
N stage	N0	37	8	29	**0.00**	22	15	**0.00**	35	12	**0.00**
	N1	24	13	11		6	18		10	14	
	N2	3	2	1		0	3		0	3	
M stage	M0	59	18	41	**0.00**	27	32	0.2	35	24	**0.01**
	M1	5	5	0		1	4		0	5	
LVI	No	35	13	22	0.8	18	17	0.1	22	13	0.1
	Yes	29	10	19		10	19		13	16	
Dukes	A	10	3	7	**0.00**	8	2	**0.00**	6	4	**0.00**
	B	26	3	23		14	12		20	6	
	C	23	12	11		5	18		9	14	
	D	5	5	0		1	4		0	5	
	A-B	35	6	29	**0.00**	22	13	**0.00**	25	10	**0.00**
	C-D	29	17	12		6	23		10	19	
Relapse	No	44	12	32	**0.03**	22	22	0.1	25	19	0.6
	Yes	20	11	9		6	14		10	10	
Alive	Dead	35	14	21	0.4	13	22	0.2	14	21	**0.01**
	Survived	29	9	20		15	14		21	8	

Data are presented as frequency (percentage) and/or mean (M) ± standard deviation (SD). Student’s *t*-test, Chi-square, and Fisher’s Exact test were used. Bold values indicate a statistically significant *p*-value below 0.05. T: tumor, N: lymph node, M: metastasis (distant); LVI: lymphovascular emboli, OS: overall survival, Rt: Right, Lt: Left, NC: neoplastic cells, IIC: infiltrating immune cell.

**Table 4:** Association of combined expression patterns of PDL-1 in NC and IIC and clinicopathological prognostic factors of colon adenocarcinomas.

Variable			PDL1				*p*-Value
			NC−/IIC−	NC+/IIC−	NC−/IIC+	NC+/IIC+	
Number			18	17	10	19	
Age (y)	≤55	29	7	7	5	10	0.8
	>55	35	11	10	5	9	
Sex	F	29	8	8	6	7	0.7
	M	35	10	9	4	12	
Laterality	RT	52	13	15	8	16	0.6
	LT	12	5	2	2	3	
Grade	G1	9	5	1	0	3	0.3
	G2	38	10	11	8	9	
	G3	17	3	5	2	7	
T stage	T1	5	1	0	1	3	0.5
	T2	15	6	3	2	4	
	T3	40	10	13	6	11	
	T4	4	1	1	1	1	
	T1-2	17	7	3	3	4	0.4
	T3-4	47	11	714	7	15	
N stage	No	37	15	10	7	5	**0.002**
	N1	24	3	7	3	11	
	N2	3	0	0	0	3	
M stage	No	59	18	17	9	15	**0.05**
	Yes	5	0	0	1	4	
LVI	No	35	12	10	6	7	0.3
	Yes	29	6	7	4	12	
Dukes	A	10	5	1	3	1	**0.001**
	B	26	11	9	3	3	
	C	23	2	7	3	11	
	D	5	0	0	1	4	
	A-B	35	16	9	6	4	**0.001**
	C-D	29	2	8	4	15	
Relapse	No	44	15	10	7	12	0.4
	Yes	20	3	7	3	7	
Alive	Dead	35	7	7	6	15	0.05
	Survived	29	11	10	4	4	

Data are presented as frequency (percentage). Chi-square and Fisher’s Exact tests were used. Bold values indicate a statistically significant *p*-value below 0.05. T: tumor, N: lymph node, M: metastasis (distant); LVI: lymphovascular invasion, OS: overall survival, RT: Right, LT: Left, NC: neoplastic cells, IIC: infiltrating immune cells.

**Table 5:** Correlation between IIC PD1, NC PDL1, IIC PDL1, combined PDL1, and BRAF protein expression in colon adenocarcinomas.

			BRAF Protein Score	BRAF Protein Positivity	IIC PD1	NC PDL1	IIC PDL1	PDL1gp
	BRAF protein Score	Correlation Coefficient	1.000	0.966 **	−0.124	0.181	0.139	0.191
		Sig. (2-tailed)	.	**0.000**	0.329	0.152	0.274	0.132
		N	64	64	64	64	64	64
	BRAF protein Positive	Correlation Coefficient	0.966 **	1.000	−0.135	0.190	0.172	0.222
		Sig. (2-tailed)	**0.000**		0.289	0.133	0.174	0.078
		N	64	64	64	64	64	64
	IIC PD1	Correlation Coefficient	−0.124	−0.135	1.000	0.062	−0.234	−0.172
		Sig. (2-tailed)	0.329	0.289	.	0.629	0.063	0.174
		N	64	64	64	64	64	64
	NC PDL1	Correlation Coefficient	0.181	0.190	0.062	1.000	0.170	0.591 **
		Sig. (2-tailed)	0.152	0.133	0.629	.	0.179	**0.000**
		N	64	64	64	64	64	64
	IIC PDL1	Correlation Coefficient	0.139	0.172	−0.234	0.170	1.000	0.895 ^**^
		Sig. (2-tailed)	0.274	0.174	0.063	0.179	.	**0.000**
		N	64	64	64	64	64	64
	PDL1gp	Correlation Coefficient	0.191	0.222	−0.172	0.591 **	0.895 **	1.000
		Sig. (2-tailed)	0.132	0.078	0.174	**0.000**	**0.000**	.
		N	64	64	64	64	64	64

Data are presented as frequency (percentage). ** A bivariate Spearman correlation test was used. Bold values indicate a statistically significant *p*-value below 0.05. NC: neoplastic cells, IIC: infiltrating immune cells, gp: group.

**Table 6:** Cox proportional hazard regression analysis of overall and disease-free survival among patients with colon adenocarcinomas.

	OS				DFS			
	Exp(B)	95.0% CI for Exp(B)		Sig.	Exp(B)	95.0% CI for Exp(B)		Sig.
		Lower	Upper			Lower	Upper	
Age (>55 vs ≤55)	2.343	0.660	8.320	0.188	1.240	0.509	3.022	0.636
Sex	1.887	0.609	5.842	0.271	1.931	0.879	4.244	0.101
Location (left vs. right)	433.2	1.437	130573.113	**0.037**	11.139	0.340	364.5	0.176
Grade (G3 vs G1/2)	0.512	0.180	1.461	0.211	0.589	0.313	1.108	0.100
T stage	11.482	1.491	88.393	**0.019**	0.947	0.369	2.431	0.909
LN stage	0.815	0.068	9.822	0.872	0.547	0.096	3.131	0.498
Metastasis	0.184	0.004	8.693	0.390	0.940	0.072	12.263	0.962
LVI	2.503	0.703	8.905	0.157	1.272	0.446	3.627	0.653
Dukes Staging	0.810	0.100	6.536	0.843	0.831	0.205	3.364	0.795
BRAF Score	0.074	0.003	1.643	0.100	1.326	0.489	3.591	0.579
BRAF Positive	11.405	0.076	1711.9	0.341	0.512	0.060	4.381	0.541
PD1 stroma	0.233	0.060	0.903	**0.035**	1.056	0.333	3.352	0.926
PDL1 tissue	3.250	1.088	9.713	**0.035**	2.241	1.003	5.006	**0.049**
PDL1stroma	0.380	0.102	1.411	0.148	1.131	0.480	2.668	0.778

Bold values indicate a statistically significant *p*-value < 0.05. OS, overall survival; DFS, disease-free survival, T: tumor, LN: lymph node, LVI: lymphovascular invasion, CI: confidence interval, PD1: programmed death 1, PDL1: programmed death ligand 1.

## Data Availability

The data sets generated and/or analyzed during the current study are available from the corresponding author on reasonable request.
